# Evolution of ethylene as an abiotic stress hormone in streptophytes

**DOI:** 10.1016/j.envexpbot.2023.105456

**Published:** 2023-10

**Authors:** Bram Van de Poel, Jan de Vries

**Affiliations:** a Molecular Plant Hormone Physiology lab, Division of Crop Biotechnics, Department of Biosystems, University of Leuven, Willem de Croylaan 42, 3001 Leuven, Belgium; b KU Leuven Plant Institute (LPI), University of Leuven, Kasteelpark Arenberg 31, 3001 Leuven, Belgium; c University of Goettingen, Institute for Microbiology and Genetics, Department of Applied Bioinformatics, Goldschmidtstr. 1, 37077 Goettingen, Germany; d University of Goettingen, Campus Institute Data Science (CIDAS), Goldschmidstr. 1, 37077 Goettingen, Germany; e University of Goettingen, Goettingen Center for Molecular Biosciences (GZMB), Department of Applied Bioinformatics, Goldschmidtstr. 1, 37077 Goettingen, Germany

**Keywords:** Plant evolution, Plant terrestrialization, Ethylene, Phytohormones, Signaling cascades, Plant physiology, Abiotic stress, Streptophyte algae

## Abstract

All land plants modulate their growth and physiology through intricate signaling cascades. The majority of these are at least modulated—and often triggered—by phytohormones. Over the past decade, it has become apparent that some phytohormones have an evolutionary origin that runs deeper than plant terrestrialization—many emerged in the streptophyte algal progenitors of land plants. Ethylene is such a case. Here we synthesize the current knowledge on the evolution of the phytohormone ethylene and speculate about its deeply conserved role in adjusting stress responses of streptophytes for more than half a billion years of evolution.

## Highlights

• Ethylene is a major abiotic stress hormone in plants and was key in plant evolution.
• The ethylene synthesis and signaling capacity is highly conserved in plants.
• Streptophyte algae acquired functional ethylene pathways for stress adaptation.
• Ethylene has been key for abiotic stress tolerance during plant terrestrialization.
• Photosynthesis and chloroplast control by ethylene is evolutionary conserved.

## 1 A current account on studying the evolution of land plants and streptophyte algae

**Fig. 1:**
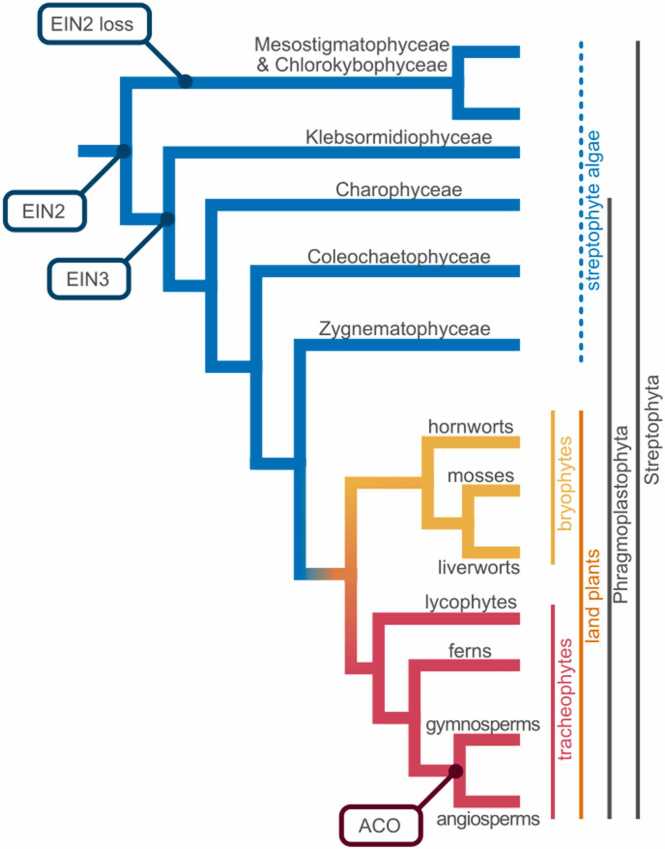
Streptophyte diversity and the evolution of ethylene signaling. The cladogram shows the phylogenetic relationship within the monophylum Streptophyta, consisting of the paraphyletic grade streptophyte algae (blue) and the land plants that are split into the non-vascular bryophytes (yellow) and vascular tracheophytes (red). Land plants (orange) form together with the three streptophyte algal classes Zygnematophyceae, Coleochaetophyceae, and Charophyceae the monophylum Phragmoplastophyta. Projected onto the cladogram is which genes of the ethylene biosynthesis and signaling machinery likely were present in which most recent common ancestor. The topology of the cladogram is based on (Wickett et al., 2014; One Thousand Plant Transcriptomes Initiative, 2019).

Our planets’ surface is covered by a monophyletic group of macroscopic photosynthetic eukaryotes: the Embryophyta, often simply called land plants. Land plants emerged from algal progenitors a little more than 500 million years ago in a fateful event that is circumscribed as plant terrestrialization (Morris et al., 2018, de Vries and Archibald, 2018, Fürst-Jansen et al., 2020). Over the last decade, a series of seminal phylogenomic studies have shed light on the relationships within land plants and between land plants and their algal relatives (Fig. 1).

The clade of Embryophyta is nested within the clade of Streptophyta. The Streptophyta consist of (i) the land plants, which are a dichotomy of the likely monophyletic Bryophyta (non-vascular plants) and the Tracheophyta (vascular plants), and (ii) the paraphylum of streptophyte algae. Among these streptophyte algae, the Zygnematophyceae are the algal sister lineage to land plants (Wickett et al., 2014, Puttick et al., 2018; One Thousand Plant Transcriptomes Initiative, 2019) (Fig. 1).

In recent years, major progress has been made on our understanding of the closest algal relatives of land plants, foremost propelled by functional genomic work. We currently consider the Zygnematophyceae to consist of five major orders, three of which contain both filamentous and unicellular algae (Hess et al., 2022). Genomes have been sequenced for representatives from four of these orders: *Spirogloeae muscicola* (Cheng et al., 2019) of the order Spirogloeales, *Penium margaritaceum* (Jiao et al., 2020) and *Closterium* sp. in a species complex of *C. peracerosum–strigosum–littorale* (Sekimoto et al., 2023) of the order Desmidiales, *Mesotaenium endlicherianum* SAG 12.97 (Cheng et al., 2019) of the order Serritaeniales, and four genomes of *Zygnema*, *Z. circumcarinatum* and *Z.* cf. *cylindricum* SAG 698–1a (Feng et al., 2023). That said, several studies salient to our understanding of Zygnematophyceae have been carried out on non-genome sequenced representatives, such as *Mougeotia* (Regensdorff et al., 2018, Fürst-Jansen et al., 2022, Permann et al., 2021), *Spirogyra* (Ju et al., 2015, Van de Poel et al., 2016, de Vries et al., 2020, Permann et al., 2022, Pfeifer et al., 2022) and diverse strains of *Zygnema* (Rippin et al., 2017, Rippin et al., 2019).

To trace the evolution of important embryophytic traits, it is not enough to look at the closest algal relatives. A comparative and complementing approach is needed. Indeed, when the genome of *Klebsormidium nitens* was released, it made a splash due to the series of homologs that it shared for several molecular pathways that were initially considered specific to land plants, foremost homologs for the biosynthesis of and signaling by phytohormones (Hori et al., 2014). A similar pattern can be seen for (i) other specialized metabolic pathways such as phenylpropanoid biosynthesis (de Vries et al., 2017, de Vries et al., 2021, Jiao et al., 2020; Rieseberg et al., 2023) (ii) and to a certain degree even in the genome of the more divergent streptophyte alga *Chlorokybus melkonianii* (Wang et al., 2020; for naming, see Irisarri et al., 2021).

In this article, we focus on the deep evolution of one of these traits once considered embryophyte-specific: the phytohormone ethylene and its ties to abiotic stress signaling.

## 2 Evolution of ethylene biosynthesis

The capacity to produce ethylene gas as a biomolecule has been achieved by organisms from across the tree of life. Ethylene biosynthesis is well known to occur in land plants, where ethylene executes the role of an important phytohormone. But also diverse bacteria, archaea and fungi have the capabilities to produce ethylene gas. In some rare cares, ethylene production has been recorded for amoebozoa, diatoms and even mammalian cells (Van de Poel et al., 2014, Papon and Binder, 2019). Yet the true biochemical nature of these observations remains obscure.

Given the fact that ethylene synthesis is not restricted to a sub-group of lifeforms, one can question whether the capacity to produce ethylene has been acquired multiple times by different species through either parallel evolution or alternatively through convergent evolution. Furthermore, it is also possible that due to million years of cohabitation, distantly related species might have exchanged genetic material and that the capacity to produce ethylene evolved through multiple horizontal gene transfer events that bridge lineage boundaries and even the eukaryote-prokaryote divide, followed by lineage-specific divergent evolution, diversification, and specialization of the biochemical and molecular pathways (Soucy et al., 2015).

**Fig. 2:**
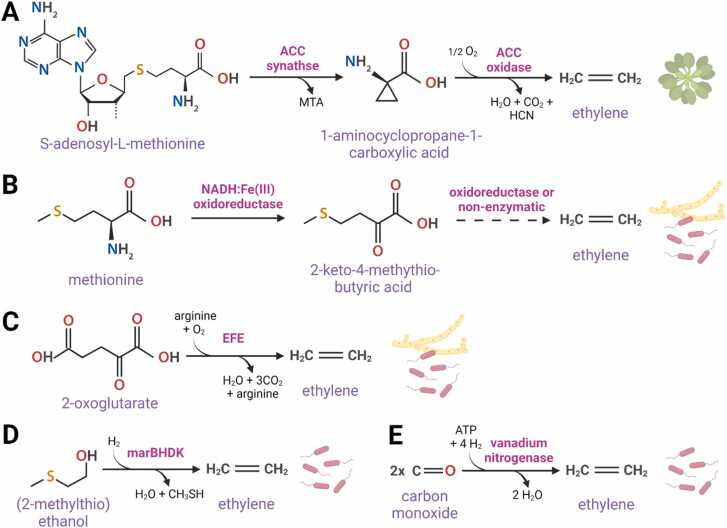
Ethylene is produced through different biochemical pathways by different life forms (plants, fungi and bacteria). (A) Plant ethylene biosynthesis pathway using 1-aminocyclopropane-1-carboxylic acid (ACC) as substrate. (B) Bacterial and fungal ethylene biosynthesis pathway using 2-keto-4-methylthiobutyric acid (KMBA) as substrate. (C) Bacterial and fungal ethylene biosynthesis pathway using 2-oxoglutarate as substrate. (D) Anaerobic bacterial ethylene biosynthesis pathway using (2-methylthio)ethanol as substrate by methylthio-alkane reductase (marBHDK). (E) Bacterial ethylene biosynthesis pathway using carbon monoxide as substrate. Abbreviations: MTA, methylthio-adenosine; HCN, hydrogencyanide; CH_3_SH, methanethiol.

Several ethylene biosynthesis pathways have been described (Fig. 2). Seed plants produce ethylene using 1-aminocyclopropopane-1-carboxylic acid (ACC) as substrate (Adams and Yang, 1979) and two dedicated enzymes. First, ACC is formed by ACC-synthase (ACS) from S-adenosyl-methionine (SAM) and subsequently converted into ethylene gas by ACC-oxidase (ACO) using molecular oxygen as co-substrate and vitamin C and CO_2_ as activator (Houben et al., 2019, Pattyn et al., 2020) (Fig. 2). Interestingly, non-seed plants and red as well as green algae seem to use a different ethylene biosynthesis pathway, as homologous genes of *ACO* have not been retrieved in their sequenced genomes (Kawai et al., 2014, Li et al., 2022). Furthermore, radiolabeled ACC feeding experiments with several non-seed plants demonstrated that ACC is not effectively converted into ethylene (Chernys and Kende, 1996, Osborne et al., 1996), suggesting another substrate for ethylene synthesis exists in non-seed plants. However, some papers reported that ACC feeding can lead to some increase in ethylene production in glaucophyte algae (*Cyanophora paradoxa*; Genot et al., 2023), red algae (*Pterocladiella capillacea* and *Gelidium arbuscula*; García-Jiménez and Robaina, 2012; Garcia-jimenez et al., 2013), chlorophyte algae (*Acetabularia mediterranea*, *Ulva intestinalis* and *Haematococcus pluvialis*; Vanden Driessche et al., 1988; Maillard et al., 1993; Plettner et al., 2005) and streptophyte algae (*Spirogyra pratensis*; Ju et al., 2015), indicative that either these species contain a distant ACO homolog or an alternative dioxygenase enzyme with substrate promiscuity for ACC. Recently, an ancestral clade of functional fungal ACO homologs was identified, of which some might have been transferred to plants via horizontal gene transfer (Li et al., 2023).

Bacteria and fungi can also produce ethylene but via different biochemical pathways (Fig. 2). Some bacteria (e.g. *Pseudomonas syringae*; Nagahama et al., 1991; Weingart and Völksch, 1997) and fungi (e.g. *Penicillium digitatum*, *Fusarium oxysporum*; Young et al., 1951; Hottiger and Boller, 1991) produce ethylene from 2-oxoglutarate (= α-ketoglutarate) and arginine using the dedicated Ethylene Forming Enzyme (EFE) in the presence of oxygen and iron (Eckert et al., 2014; Fig. 2). Other microorganisms produce ethylene from 2-keto-4-methylthiobutyric acid (KMBA; also named 2-methylthio-2-oxobutanoate) which is formed from methionine by a NADH:Fe(III)EDTA oxidoreductase (Shipston and Bunch, 1989), and is believed to be subsequently decomposed non-enzymatically into ethylene gas by photo-oxidation or hydroxyl radicals (Chagué et al., 2002). The KMBA-dependent ethylene synthesis pathway has been described in some bacteria (e.g. *Escherichia coli*, *Aeromonas* B12E; Billington et al., 1979; Mansouri and Bunch, 1989) and fungi (e.g. *Botrytis cinerea*; Chagué et al., 2002). Some aerobic N-fixing (diazotrophic) bacteria, such as *Azotobacter vinelandii*, are able to convert 100 % carbon monoxide (CO) into ethylene gas using a vanadium nitrogenase (V; Lee et al., 2010; Hu et al., 2011). Recently, another nitrogenase-like protein (methylthio-alkane reductase; *marBHDK*) from a diversity of terrestrial and aquatic photosynthetic alphaproteobacteria (e.g. *Rhodospirillum rubrum*, *Rhodospeudomonas palustris*; North et al., 2017) was discovered, and is able to anaerobically synthesize methionine from the sulfur-compounds (2-methylthio)ethanol, thereby releasing ethylene gas into the environment (North et al., 2020).

## 3 Evolution of ethylene signaling

The capacity to sense ethylene by means of a bioreceptor is ancient. Extant photosynthetically active cyanobacteria (e.g. *Synechocystis* strain PCC 6803, *Geitlerinema sp.* PCC 7105 and several others; Wang et al., 2006; Allen et al., 2019) have an ethylene receptor (e.g. slr1212) that resembles the ethylene receptor (ETR) well studied in the model plant *Arabidopsis thaliana* (Kaneko et al., 1996, Rodríguez et al., 1999). This suggests that sensing ethylene must have a cyanobacterial origin, far predating the endosymbiotic origin of plastids roughly 2 billion years ago (for timing, see Strassert et al., 2021), and was likely acquired by the archaeplastidial host of plastids (and thus ultimately land plants) through endosymbiotic gene transfer (Mount and Chang, 2002). Interestingly, cyanobacteria that harbor an ethylene receptor do not seem to produce ethylene themselves (Guerrero et al., 2012, Ungerer et al., 2012) — with the exception of a filamentous cyanobacterium *Hapalosiphon* reported by Huang and Chow (1984). This suggests that cyanobacteria likely sense environmental ethylene (Lacey and Binder, 2016), a chemical signal produced by fires and radiation-induced decay of organic matter (Papon and Binder, 2019).

The ethylene receptor of seed plants has resemblance to a bacterial two-component system that typically senses external environmental signals, consisting of a sensory, transmitter and receiver domain (Chang et al., 1993). Genomes of numerous species ranging from charophyte green algae to seed plants all have homologous genes that code for ethylene receptor proteins (Cheng et al., 2019, Ju et al., 2015, Van de Poel et al., 2014) (Fig. 1). While some genomes of chlorophyte algae contain candidate ETR homologs (e.g. *Coelastrella vacuolata* (Shetty et al., 2021); *Coccomyxa subellipsoidea* (Blanc et al., 2012)), others only have a partial sequence (e.g., *Ostreococcus tauri* (Derelle et al., 2006) and *Chlamydomonas reinhardtii* (Merchant et al., 2010)), which might suggest some chlorophyte lineages lost their capacity to sense ethylene. Indeed, clear examples for the loss of ethylene signaling exists. The marine seagrass *Zostera*, which transitioned 65 million years ago from a terrestrial lifestyle to an aquatic one, has lost the genes coding for ethylene receptors and other ethylene signaling and biosynthesis components (Golicz et al., 2015, Olsen et al., 2016). Yet, this is not an inevitable consequence of an aquatic lifestyle, as (a) the aforementioned *Chlamydomonas reinhardtii* is a soil-dweller and (b) we will outline below cases of aquatic streptophyte algae that have a full homologous chassis for ethylene signaling.

Candidate ethylene receptor homologous sequences have also been retrieved in genomes of prokaryotes (proteobacteria), terrestrial and aquatic fungi, free-living amoebas, protists, diatoms and even some opisthokont protists (Mesomycetozoa; Wang et al., 2006; Papon and Binder, 2019). However, the actual biological functionality of the ethylene receptor has only been shown for the cyanobacterium *Synechocystis* PCC6803 (Lacey and Binder, 2016), the Zygnematophyceae *Spirogyra pratensis* (Ju et al., 2015, Ju et al., 2015), the moss *Physcomitrium patens* (Yasumura et al., 2012) and many representative species of angiosperms, corroborating the idea that the ethylene sensing system of land plants emerged in their streptophyte algal ancestors prior to land colonization.

Components of the ethylene signaling pathway as known in seed plants, were likely assembled during the evolution of phragmoplastophytic streptophyte algae (Fig. 1). Ju et al. (2015) showed that several components of the alga *Spirogyra pratensis* that operate downstream of the ethylene receptor are functional when ectopically expressed in corresponding *Arabidopsis* mutants. For the known ethylene signaling pathway of Arabidopsis, the ethylene receptor interacts with a RAF-like signaling kinase CONSTITUTIVE TRIPLE RESPONSE 1 (CTR1; Kieber et al., 1993) that phosphorylates the downstream endoplasmic reticulum-bound protein ETHYLENE INSENSITIVE 2 (EIN2; Alonso et al., 1999) in the absence of ethylene to targets it for degradation (Qiao et al., 2009, Ju et al., 2012). CTR1 homologs are widespread in eukaryotes, but not in prokaryotes (Kieber et al., 1993). The Arabidopsis EIN2 is similar to an Nramp metal ion transporters, which are found in almost every life form (Nevo and Nelson, 2006). However, homologs of EIN2 appear to be absent in the genomes of chlorophytes and those streptophyte algal lineages most distantly related to land plants (Chlorokybophyceae and Mesostigmatophyceae; Wang et al., 2020). In contrast, EIN2 homologs occur in the genomes Zygnematophyceae and the Charophyceae *Chara braunii* (Wang et al., 2015; Nishiyama et al., 2018; Jiao et al., 2020). In Arabidopsis, CTR1 phosphorylates EIN2 in the presence of ethylene, and this leads to EIN2 being cleaved so that the C-terminal part migrates to the nucleus (Ju et al., 2012, Qiao et al., 2012, Wen et al., 2012) to activate the master transcription factor EIN3 (Chao et al., 1997). Sequences of EIN3 and homologs (EIN3-LIKE; EILs) are present in genomes of land plants, but are also retrieved in genomic and transcriptomic data of several streptophyte algae (Cheng et al., 2019, de Vries et al., 2018a, Hori et al., 2014, Ju et al., 2015, Mao et al., 2022), but not in the streptophyte algal lineages Chlorokybophyceae and Mesostigmatophyceae and chlorophyte algae (Mao et al., 2022, Wang et al., 2015, Wang et al., 2020). Therefore, based on sequence information and a few functional studies, we can conclude that the full ethylene signaling pathway was assembled in the common ancestor of Zygnematophyceae and land plants, and thus fully operational during the colonization of land by plants.

## 4 Abiotic stress responses across streptophyte diversity

**Fig. 3:**
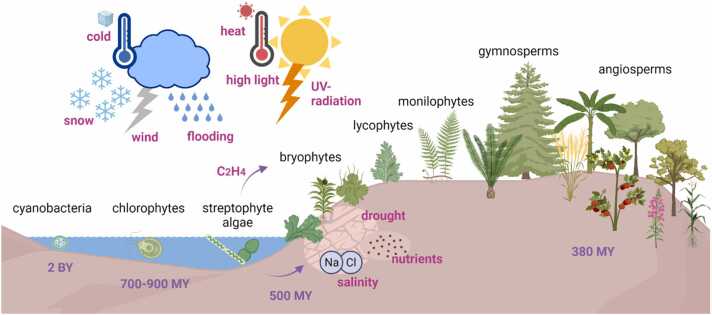
Overview of plant evolution and the crucial transition from an aquatic to a terrestrial lifestyle. Ancestors of fresh-water streptophyte algae conquered land roughly 500 million years ago after the acquirement of ethylene as a functional plant hormone (amongst others), to enable responses towards to a diversity of abiotic stressors inherent to this habitat change.

Among streptophytes, not only land plants but also diverse streptophyte algae dwell in terrestrial habitats (Lewis and McCourt, 2004, Fürst-Jansen et al., 2020, Permann et al., 2022). Transitioning from an aquatic to a terrestrial habitat comes with a range of stressors that are particular—and/or particularly severe—on land (Fig. 3). These included new biotic interactions (both challenging and beneficial), which have been the focus of other reviews (de Vries et al., 2018b, Delaux and Schornack, 2021); here, we solely focus on abiotic challenges.

Abiotic challenges on land have a two-pronged dynamic: rapidity and severity. They include rapid and drastic temperature shifts, high irradiance, water availability and many more. Land plants have a broad repertoire for responding to abiotic challenges. To put this in numbers: in Arabidopsis, 4950 genetic loci are "response to abiotic stimulus" (based on TAIR, Lamesch et al., 2012). In contrast, aquatic angiosperms have far fewer stress-related genes. The marine monocot *Zostera marina* has only 144 differentially expressed transcripts associated with “environmental adaptation” pathways when exposed to a variety of abiotic stressors (Kong et al., 2014). The transcriptomic comparison of aquatic and terrestrial *Ranunculus* species revealed that only 14 genes were associated with the adaptive transition from terrestrial to aquatic habitats (Chen et al., 2015). Nonetheless, facing abiotic challenges is not limited to land plants—especially given that many streptophyte algae (and other algal groups) live on land. It is thus not surprising to find conserved as well as divergent response mechanisms to stressors in land plants and their algal relatives.

Some of the most palpable responses to abiotic challenges is the production of specialized compounds. Through different manners, these act as signaling compounds and/or directly mitigate the adverse effects of abiotic stressors. Several of these pathways have a shared evolutionary origin in streptophyte algae (Dadras et al., 2023a, Maeda and Fernie, 2021). Examples include homologs for a shared genetic chassis and in specific cases even the measurable production of phenolic compounds, different carotenoid, and other isoperenoid(-derived) compounds (de Vries et al., 2021, Jiao et al., 2020, Pichrtová et al., 2013, Remias et al., 2012; Davies et al., 2022). That being said, some pathways are clearly divergent, including the UV protectants Mycosporine-like amino acids formed by streptophyte algae such as *Klebsormidium* (Karsten and Holzinger, 2014b, Karsten et al., 2014a; Hartmann et al., 2020). Similar recurrent patterns occur on the protein level. LATE EMBRYOGENESIS ABUNDANT (LEA) is induced across diverse streptophyte algae and stressors (Rippin et al., 2017, de Vries et al., 2018a). LEAs are a large classical stress responsive proteins known from land plants (Hundertmark et al., 2008). Similarly, photosynthesis-associated changes frequently occur—more on this below.

A shared chassis for downstream responses begs the question of whether there are similarities to be found upstream too. Some attention has been given to certain transcription factors, such as MYB whose regulation of genes responsible for specialized metabolism is conserved across land plants (Albert et al., 2018). In land plants, upstream regulatory processes are either directly governed and/or modulated by phytohormones (Scheres and van der Putten, 2017). This also holds true for ethylene in general, as well as in the particular case of the interplay between ethylene, stress, and MYBs (Cheng et al., 2013, An et al., 2018, Zhang et al., 2019). Another famous example is abscisic acid. Here, the canonical signaling cascade is based on a chain of negative regulation: ABA binds to receptors of the PYRABACTIN RESISTANCE(-LIKE)/REGULATORY COMPONENT OF ABA RECEPTOR (PYR/PYL/RCAR) family that inhibit PROTEIN PHOSPHATASE C proteins that would otherwise inhibit the activators of the downstream targets, the SUCROSE NONFERMENTING 1-RELATED PROTEIN KINASE2 (SnRK2; Ma et al., 2009; Park et al., 2009; Rubio et al., 2009; Vlad et al., 2009; Umezawa et al., 2009; Nishimura et al., 2010; Cutler et al., 2010). Some Zygnematophyceae, including *Zygnema* and *Mesotaenium*, have homologs for the entire chassis needed for ABA-mediated signaling (Cheng et al., 2019, de Vries et al., 2018a, Feng et al., 2023). That said, investigations into the function of the PYL–PP2C module of *Zygnema* have shown that while PYL does inhibit the activity of PP2Cs, it does so in an ABA-independent manner (Sun et al., 2019). This illustrates both the deep evolutionary origin of the connections in the cascade and how small differences in the input can alter the function of an entire molecular chassis (see also discussions in Fürst-Jansen et al., 2020).

In case of ethylene, it however appears that it acts as a bona fide stress hormone since before the dawn of land plants.

## 5 The evolutionary role of ethylene in abiotic stress responses

Despite the pleiotropy of developmental responses controlled by ethylene in seed plants, a common function of ethylene in all plants that produce and/or sense ethylene seems its role in mediating stress responses (Fig. 3). Indeed, stress-specific ethylene responses have been characterized in cyanobacteria. These photosynthetic prokaryotes do not produce ethylene themselves, but responds likely to externally produced ethylene, hence the role of ethylene in environmental sensing. External ethylene can be produced by other microorganisms or abiotically by incomplete combustion and decay of organic matter by light (Papon and Binder, 2019). It was shown that the cyanobacteria *Synechocystis* sp. PCC 6803 and *Geitlerinema* sp. PCC 7105 responded to ethylene by stimulating their phototaxis towards the light (Lacey and Binder, 2016, Allen et al., 2019). Disruption of the function of the ethylene receptor SynETR1, led to a larger movement towards the light source independent of ethylene, suggesting that the ethylene receptor is a negative regulator of ethylene responses in *Synechocystis*, similar as in angiosperms (Lacey and Binder, 2016). Besides phototaxis, ethylene signaling also stimulates pili formation and cell surface sugar moieties, that enhances biofilm formation and cell sedimentation (Lacey and Binder, 2016, Lacey et al., 2018), traits cyanobacteria use to respond to environmental cues, including light perception. Ethylene also stimulates growth in a dose dependent way, and alters the fatty acid composition of *Synechocystis* (Le Henry et al., 2017). All these bacterial ethylene-mediated responses indicate that already the cyanobacterial progenitors of plastids might have had the capacity to sense external ethylene as a signaling compound to properly respond to abiotic stressors.

A clear role for ethylene in mediating abiotic stress was also observed in the zygnematophye *Spirogyra pratensis*, which has the capacity to produce ethylene and a fully functional ethylene signaling pathway, as known in seed plants (Ju et al., 2015). *Spirogyra pratensis* responds to different abiotic stress conditions by means of cell elongation, a response previously linked to ethylene, and which could be inhibited by 1-MCP (1-methylcyclopropane; a potent inhibitor of ethylene signaling; Van de Poel et al., 2016). Furthermore, an ethylene treatment lead to the differential expression of a large group of genes annotated to be involved in abiotic stress responses (Van de Poel et al., 2016). The role of ethylene in regulating abiotic stress in streptophyte algae was corroborated by the detection of differentially expressed ethylene-related genes in *Klebsormidium*, belonging to the Klebsormidiophyceae that are sister to the phragmoplastophytes, during desiccation (Holzinger and Becker, 2015), another six streptophyte algae in response to cold stress (de Vries et al., 2018a), and the zygnematophyte *Mesotaenium endlicherianum* upon light and temperature cues (Dadras et al., 2023b).

There is little evidence about the role of ethylene in directing abiotic stress resilience in non-seed plants such as bryophytes and lycophytes. Recently, it was shown that ethylene signaling and biosynthesis is key to tolerate heat, salt, nutrient and light stress in the liverwort *Marchantia polymorpha* (Bharadwaj et al., 2022). Phosphate starvation also induced transcriptional changes of ethylene-related genes in Marchantia (Rico-resendiz et al., 2020). Submergence tolerance was observed to be mediated by ethylene signaling in the moss *Physcomitrium patens* (Yasumura et al., 2012). These few reports corroborate the notion that the ancestor of setaphytes (and very likely all bryophytes) already used ethylene as a signaling molecule to adapt to a change in environmental conditions. Together with the knowledge that also streptophyte algae use ethylene to respond to abiotic stresses, we conclude that ethylene is a conserved signal upon the dramatic change of environmental factors (e.g. heat, drought, light, nutrient stress). Therefore, a functional ethylene pathway was likely present in a phragmoplastophytic algal progenitor of land plants; it is conceivable that ethylene signaling has aided the earliest land plants more than 500 million years ago in coping with harsh abiotic stressors they faced during the conquest of land (Fig. 3).

## 6 Ethylene and abiotic stress: deep evolutionary roots of directing plant photosynthesis

**Fig. 4:**
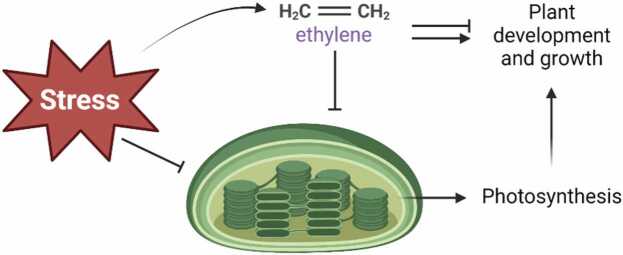
The intricate and conserved relation between abiotic stress, ethylene and the plastid. Abiotic stress impacts plastid performance and photosynthesis, directly or indirectly through ethylene as an important growth regulator. The role of ethylene in steering plant photosynthesis in evolutionary distant species ranging from cyanobacteria to angiosperms suggests fine-tuning photosynthesis is one of ethylene’s prime function in plants. The plastid plays a central role in mediating both stress signaling and ethylene responses, a feature conserved throughout plant evolutionary history.

Mediating the responses to abiotic stress is intertwined with plastid biology—the plastid can be considered an environmental sensor and hub of a signaling network in the plant cell (Kleine et al., 2021) (Fig. 4). Ethylene is likely woven into this signaling network: one of the physiological processes regulated by ethylene is photosynthesis (Ceusters and Van de Poel, 2018) (Fig. 4). The adjustment of photosynthesis by ethylene is well-studied in angiosperms such as Arabidopsis (Grbic and Bleecker, 1995), tomato (Woodrow and Grodzinski, 1989), rice (Gautam et al., 2022) and wheat (Taylor and Gunderson, 1986). Interestingly, ethylene also exerts a controlling function over photosynthesis in streptophyte algae and non-seed plants. In the zygnematophyte *Spirogyra pratensis*, it was shown that ethylene downregulates photosynthesis and represses many genes coding for components of the photosynthetic machinery (Van de Poel et al., 2016). It was also observed that chlorophyll content was lower in *Spirogyra* after ethylene treatment (Van de Poel et al., 2016), a phenotype shared with Arabidopsis (Zacarias and Reid, 1990). In the liverwort *Marchantia polymorpha*, chlorophyll content was lower in an *ein3* mutant, suggesting ethylene is involved in regulating normal chlorophyll levels and thus photosynthesis (Bharadwaj et al., 2022). In the cyanobacterium *Synechocystis*, ethylene enhances the levels and quantum efficiency of photosystem II (PSII), and hence stimulates photosynthesis, probably to optimize light perception (Lacey and Binder, 2016, Le Henry et al., 2017).

In summary, ethylene exerts either a positive or a negative effect on photosynthesis in evolutionarily distant species ranging from cyanobacteria to angiosperms. Therefore, we conclude that the origin of the regulation of photosynthesis by ethylene is ancient and has been conserved during the evolution of land plants. As such, the control of photosynthesis is one of the primary and most-conserved processes governed by ethylene. This has implications on the evolution of the stress signaling network of embryophytes: the plastid is a centerpiece in the molecular network for plant acclimation (Kleine et al., 2021) and the physiological status of plastids is governed by photosynthesis. This means that ethylene-controlled processes have leverage over plant physiology through the convergence on one of its major denominators. These interconnections make it one of the major stress hormones of plants. We now postulate that ethylene produced by plants themselves in response to abiotic stress, or ethylene sensed in the environment (e.g. produced by neighboring plants, microorganisms or organic decay), provided land plants with essential means to fine-tune plant physiology in a changing environment. This was the case ∼500 million years ago in the Cambrian when land was first colonized by plants, and is still the case at present day during the late Anthropocene period when plants are forced upon climate change.

## CRediT authorship contribution statement

Both BVdP and JDV performed literature research and wrote the article.

## Declaration of Competing Interest

The authors declare the following financial interests/personal relationships which may be considered as potential competing interests: Bram Van de Poel reports financial support was provided by European Research Council. Jan de Vries reports financial support was provided by European Research Council. Bram Van de Poel reports financial support was provided by Research Foundation Flanders. Jan de Vries reports financial support was provided by German Research Foundation.
